# Rural Residents’ Awareness of Environmental Protection and Waste Classification Behavior in Jiangsu, China: An Empirical Analysis

**DOI:** 10.3390/ijerph17238928

**Published:** 2020-12-01

**Authors:** Aijun Liu, Maurice Osewe, Huixin Wang, Hang Xiong

**Affiliations:** 1 College of Economics and Management, Nanjing Agricultural University, 1 Weigang, Nanjing 210095, China; mauriceosewe@gmail.com (M.O.); wangwanghuixin@163.com (H.W.); xionghang@njau.edu.cn (H.X.); 2 China Center for Food Security Studies, Nanjing Agricultural University, 1 Weigang, Nanjing 210095, China

**Keywords:** waste classification, environmental protection, rural waste, environmental protection awareness

## Abstract

Achieving waste classification and environmental protection awareness assists in enhancing the rural ecological environment, improves the quality of the rural residents’ life, and augments the rate of reusing resources within the rural setups. In order to study the factors influencing rural waste classification and environmental protection awareness, we conducted a project among rural residents of Jiangsu, China. We used both the logistic regression model and the ordinary least squares model to achieve the goals of this paper. Similarly, we found that the households’ level of education influenced the rural residents’ decision to classify waste and to protect the environment regarding whether a household consists of a village cadre; the availability of public waste collection facilities; the distance between households and the waste collection points; whether the waste is picked, assorted, and collected locally; and the cost of waste disposal. In light of all these factors, we recommended that the authorities should increase the rural residents’ waste classification and environmental protection awareness. Also, through the local government, the national government should strengthen and sustain rural waste disposal funds. Finally, there should be stringent laws and regulations outlining the role of the rural residents regarding waste classification and environmental protection awareness.

## 1. Introduction

China has experienced fast economic development during the last several decades, a trend that has given rise to unprecedented solid waste production. Even more, China became the largest solid waste generator in 2004 and is projected to have the fastest and largest waste growth historically [1]. The authors of [2] observed that the per capita annual growth rate of solid waste generation in China ranges from 8% to 10%. In essence, a further increase in economic growth would lead to a greater increase in the rate of solid waste production. 

Currently, a great number of rural residents are unaware of the environmental protection and household waste classification. Yet, rural areas account for about 60% of mainland China. That is, the rural population totals approximately 675 million people residing in about 40,000 small towns [1]. Researchers estimate that the rural solid waste generation rate ranges from 0.25 kg to 2.1 kg per capita per day [3]. In this study, we use the term ‘rural household waste’ to describe solid waste consisting of recyclable waste, wet waste, and nonrecyclable wastes. However, in most cases, rural household waste mainly consists of kitchen waste [4,5]. Large rural population growth and increasing incomes are catalysts in increasing rural household waste production in China. Further, rural solid waste affects the environment in several ways. For instance, it contaminates air, soil, and water in rural areas, and harms the residents’ health in the long term. On the other hand, in most rural areas of China, between 30% to 60% of the solid waste is dumped [6]. This has led to rural environmental quality deterioration. For instance, the authors of [7] concluded that random dumping of solid waste in rural China contaminated about 100,000 km^2^ of the agricultural land. Equally, the undesirable handling of wastes creates harmful substances, diseases, and water pollution that jeopardize the health of the rural population. Appropriate waste disposal, which involves classification and collection is the new technique used in controlling solid waste pollution in China. For instance, Jiangsu has been promoting the collection transportation and processing system of waste treatment. Even more, the waste classification process forms the starting phase of pro-environmental behavior, a factor that enhances the rural residents’ significant ecological behavior [8,9].

Nonetheless, most of the rural residents do not use the recommended waste control practices such as classification. This could arise from several factors, such as lack of environmental protection awareness or lack of information flow among the residents. Other factors may include structural or cultural characteristics of rural residents. Besides, the ability to recycle solid waste has drastically diminished because of the increasing development of the Chinese rural socio-economy [10]. This has modelled the adverse impacts of the rural environment. As a result, rural waste management has attracted significant consideration from the Chinese government which enacted several laws and regulations strengthening waste management. China incorporated the rural solid waste into the scope of the public administration, emphasizing the need to control waste and sewages to enhance the living environments of the rural population. However, despite the efforts put in place by the government, challenges still exist, such as inherent characteristics of waste classification, lack of facilities, and administrative measures that overwhelm rural waste management [11,12,13].

Various and distinctive technical and social innovations are needed for rural solid waste management based on specific geographical regions [14]. The rural solid waste classification method is recommended to handle such challenges. This method reduces waste bulkiness and improves recycling efficiency [15]. Nonetheless, inadequate research has been conducted about factors enhancing rural waste classification and environmental protection in Chinese rural areas. Based on the available literature, studies concur that family and individual attributes influence the waste classification and environmental protection awareness. For instance, household income influences positively the rural residents’ environmentally beneficial character. Also, individuals with formal education are environmentally conscious [8]. Nonformal education, such as watching television or browsing the Internet, also offers an avenue for gaining knowledge. There is a negative relationship between age and environmental protection awareness. Older people tend to dispose of wastes without regarding the designated and classified bins [16]. On the other hand, women are more involved in waste classification compared to men [2,17].

Based on the literature and survey data collected in Jiangsu Province, China, this study offers both anecdotal and empirical evidence of factors enhancing rural waste classification and environmental protection awareness. Typically we proposed the following hypotheses:

**Hypotheses 1** **(H1).** *Rural populations’ demographic characteristics significantly influence the household waste classification behavior and environmental protection awareness. Level of education significantly and positively influences environmental protection awareness. Income has a positive and significant influence on solid waste classification behavior. Also, age significantly and negatively influences the waste classification behavior and environmental protection awareness.*

**Hypotheses 2** **(H2).** *Institutional and social variables significantly enhance solid waste classification behavior and environmental protection awareness. Solid waste picked, classified, and collected locally significantly influences the environmental protection awareness and household waste classification. The distance between the waste collection point and rural households significantly influences waste classification behavior. Imposing charges on rural waste collection can also enhance environmental protection and waste classification.*

## 2. Methods and Materials

### 2.1. Study Area

Jiangsu is located along the eastern coast of China. It has 102,600 km^2^ of land and a population of almost 80 million. Further, Jiangsu is among the leading provinces in education, tourism, technology, and finance. It is the third smallest province in China, although the most densely populated. In terms of gross domestic product (GDP) and GDP per capita, it is the second highest and highest province, respectively. For instance, the 2018 nominal GDP was more than 1.39 trillion dollars. Influenced by natural factors and economic development, Jiangsu is divided into northern, central, and southern regions. Further, considering the representative of sample cities and population size, four cities were chosen for survey. The cities included were Xuzhou and Suqian in the north, Nantong in the central, and Changzhou in the south. Xuzhou is a national complex transport hub and a central city of the Huahai economic zone. Suqian, Changzhou, and Nantong, on the other hand, are prefecture-level cities. Basic information of these four cities are summarized in Table 1.

**Figure 1:**
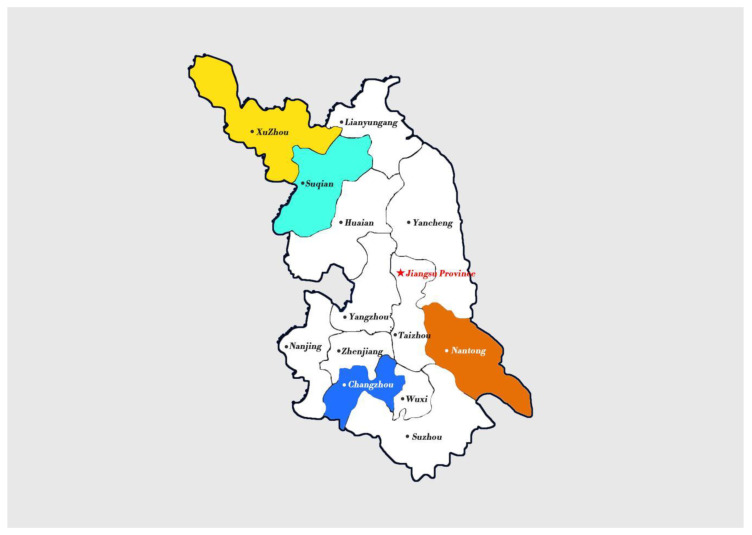
A map showing cities in Jiangsu Province.

We conducted a survey in 16 villages in the four named cities in Jiangsu. Applying a proportionate random sampling method, we selected 40 households from each village to participate. Finally, a total number of 636 respondents accepted and participated in this study. Figure 1 illustrates the map of Jiangsu Province, detailing the selected cities.

### 2.2. Questionnaire Design

The questionnaires used in this study were designed based on the existing literature [12,18], which majorly reflected on the demographic characteristics, environmental protection, public service provision, and policy institutional environment. Table 2 explains the different variables as used in the questionnaire. The family and resident characteristics, such as age, household size, educational level, and monthly household income, among others, were reflected in the first part of the questionnaire. The residents’ awareness of the environmental protection and waste classification was presented in the second part of the questionnaire, with questions such as whether waste classification is necessary and whether it is necessary to carry out environmental protection, among others. Provision of public services was the third part of the questionnaire, which contained factors such as whether the village has waste collection facilities, the distance between the households and the collection points, and whether waste collection is charged, among others. Further, policy institutional environment was the fourth part and consisted of questions such as whether there are rewards for the residents with high waste classification knowledge and whether to educate the residents about environmental protection. Waste centralized collection behavior was illustrated in part five of the questionnaire, with questions such as whether residents package waste and dispose in the right facility and the frequency of waste classification. Finally, simple waste handling behavior was the last section of the questionnaire, which highlighted factors such as whether the residents undertake waste classification and whether they recycle and sell wastes.

Also, we checked, edited, coded, and transcribed the data. We performed the statistical analysis on the 636 valid questionnaires and tested the reliability and validity. We tested for the reliability using the Cronbach’s Alpha of all the observed variables [14]. The results indicated that the Cronbach’s Alpha coefficient was 0.892. This confirmed that the overall reliability of the adjusted data is good considering the conclusions of the authors of [16], which stated that reliability is good when its coefficient is greater than 0.7. Even more, the samples with the highest score amounted to 0.31% of the total outcome.

### 2.3. Empirical Model

1. Residents’ scores about environmental protection awareness and rural waste classification behavior = F_1_ (Factors influencing the residents’ classification and awareness behavior, *i* = 1,2,3…n).

In this model, we analyzed the factors influencing the rural waste classification and environmental protection awareness in Jiangsu, China. We used three questions to reflect on the rural residents’ knowledge of environmental protection and waste classification. The questions included were: ‘Is it necessary to carry out environmental protection work?’, ‘Do you understand rural waste classification?’, and ‘Is the rural waste classification appropriate?’ On the other hand, evaluating the factors enhancing the rural residents’ environmental protection and waste classification, we embarked in answering the two hypotheses, H1 and H2. We used logistic regression method to ascertain the determinants of environmental protection awareness and waste classification among the rural residents of Jiangsu Province. The model was specified mathematically as [19]:

(1) $$p=F\left(y=1|X_{i}\right)=\frac{1}{1+e^{-y}}$$

In the equation, *p* represents the probability of the rural residents’ classification and environmental awareness decisions, and *X_i_* represents the factors influencing the residents’ classification and awareness behavior, *i* = 1,2,3 …n. To further ensure the robustness of the results, we modelled the ordinary least squares (OLS) method. The model was specified as [20]:

(2) $$y=\beta_{0}+\beta_{1}x_{1}+\beta_{2}x_{2}\cdots +\beta_{16}x_{16}+\mu$$

where, y represents the residents’ scores about environmental protection awareness and rural waste classification behavior. *x*_1_, … *x*_16_ represents the determinant variables, *β*_1_, … *β*_16_ are the regression coefficients, *β*_0_ is the constant term, and µ is the random error term.

## 3. Results

In this section, we present the descriptive results, environmental protection awareness results, and household waste classification behavior results from both the ordinary least squares model and the logistic regression model.

### 3.1. Descriptive Statistics

Table 3 represents the rural residents’ descriptive statistics. Most of the respondents were male, with a mean of 0.544, and mostly had formal high school education, with a mean of 3.785. Rural households had, on average, four members. Further, most of the households earned a monthly income ranging between RMB 5000 and RMB 7000. The mean distance between the households and the collection point was 224.68 m. The mean average of the households with the waste classification knowledge was 1.475, and the mean average of whether a waste classification was necessary was 0.974. Further, Table 3 offers an overview of the variable description and categories.

### 3.2. Logistics Regression and OLS

We used the Stata software to perform the logistic and OLS regression analysis of the rural residents. Table 4 illustrates the outcomes of both the regression models, and it can be observed that the two models almost produced the same significant variables. This implied the robustness of the data and findings. Therefore, the following variables influenced rural waste classification and environmental protection awareness significantly. Whether there was a party member or village cadre at home influenced the ecological and waste classification positively. Further, the distance between the rural households and the waste collection point was negative and significant. Also, whether the rural residents collected wastes after sorting/classifying was positive and significant. Whether the village contained waste collection facilities was significant and positive in influencing the environmental protection awareness and rural waste classification. Even more, the level of education of the residents involved in the survey was positive and significant.

Similarly, on the household waste classification behavior, we used both the logit model and OLS model. In Table 5, we have two main columns, that is, waste collection behavior and simple waste classification behavior. Regarding the waste collection behavior, factors such as gender of the household head, existence of village cadre within the household, monthly household income, availability of nonresident student in the household, knowledge of waste classification, whether waste classification is necessary, availability of public waste collection facilities, and whether to educate the residents on environmental protection significantly influenced the residents’ waste collection behavior. On the other hand, simple waste classification behavior, including whether it is necessary to undertake environmental protection work, knowledge of waste classification, whether waste classification is necessary, the distance between the waste disposal point and the households, wether garbage can(or trash pool) is cleaned regularly, environmental and waste classification awareness, and whether to educate the residents on the behavior of protecting the environment, was significantly influenced by the educational level of the household head.

## 4. Discussion

Most of the research conducted internationally on waste classification and environmental protection awareness have indicated that policy measures play a significant role in influencing the residents’ environmental beneficial behaviors [8]. Further, policy measures with a focus on rural waste classification behavior significantly enhance the residents’ attitude to classify solid wastes before disposal. In essence, training the rural residents on how to classify the solid wastes and how to become environmentally conscious influences the frequent occurrences of waste classification and environmental protection. The policymakers in China have similarly embarked in implementing several policies in line with rural waste management, particularly in Jiangsu Province, because of the increasing pressure from environmental pollution [1]. Particularly, the main policy that has been implemented in Jiangsu regarding the rural waste management and environmental protection consists of rural waste classification before disposal as well as environmental protection awareness. As a result, we sought to understand the driving factors that motivate the rural residents’ choices to adopt to these policies. We employed two empirical models, the logistic regression model and the ordinary least squares model. The overall model representation indicated a significant prediction with Prob > Ch^2^ of 0.001. Further, the coefficients of the regression model ranged between 0 and 1, a significant indicator that the models greatly explained the factors enhancing rural waste classification behavior and environmental protection awareness. As noted earlier, both models produced more consistent results.

Therefore, a rural household that has a party member or village cadre within the household was observed to classify rural waste and was also aware of the environmental protection necessities. This is in line with the second hypothesis of this paper that party members or village cadres are aware of the government policies and try to assist in their implementation. Hence, they are conscious of the environmental protection as well as rural waste classification. Further, nationally, several efforts have been put in place to promote rural household waste classification, a factor that the village cadre strengthens and that influences the rural residents to adopt and to enhance awareness [21]. This finding concurs with several other pieces of research that opine that institutional procedures or authorities improve the dissemination of information, particularly waste classification and environmental protection awareness [4,17,22].

Equally, the coefficient of the distance between the rural households and the waste collection point was significant with a negative magnitude. This is consistent with the second hypothesis of this paper. This implies that the further away the waste collection point is, the more inconvenient it is for the rural residents to classify rural waste and to protect the environment [23]. Besides, it leads to higher opportunity costs of taking time to classify the wastes and hence correctly dispose of them anywhere [3,24]. This leads to environmental pollution. The likelihood of not classifying and dumping waste diminished by 0.14% with every one-meter increase between the rural households and the collection points. This finding conforms with the assertions concluded by the authors of [5] that the distance between the rural households and the waste collection points play a major role in whether one disposes of the waste anywhere or within the stipulated areas. Further, households that are near the collection points tend to assume the classification behavior because of their proximity [12,18].

Level of education positively and significantly influenced rural waste classification and environmental protection awareness. Rural residents with higher education levels are more probable to collect, classify, and be aware of the need for environmental protection [11]. On the other hand, we assumed that lack of education diminishes the environmental awareness to the point that a rural resident might not be observant about environmental pollution and the need for awareness. According to the authors of [25], the likelihood of rural residents dumping wastes in the right point is associated with a 1% increase per additional year of education. Further, other studies observed that having environmental education improves the residents’ sense of environmental protection awareness. This also includes understanding the benefits of collecting and classifying rural wastes in their day-to-day life [13,25]. Therefore, the more learned an individual is, the higher environmental protection awareness they have.

Similarly, the villages that had public waste collection facilities were aware of the rural waste classification as well as environmental protection. Waste cans and pools help the residents in sorting the kinds of rural solid waste [26]. This implies that the environment is conserved and protected as the residents dump the available household wastes in the right waste bins after classification. Further, the availability of the waste bins within the rural areas conveniences the rural residents and improves the frequency of cleaning and classification [13]. Even more, it increases the likelihood of attending to environmental health. According to the authors of [27], when the authorities install the waste bins in the rural areas, demonstrations are often conducted. This increases public awareness of waste classification and environmental protection. These findings confirm that the availability of the waste bins in the rural setups positively and significantly influences the environmental protection awareness, as well as the rural waste classification behavior among rural households.

Picking, classifying, and collecting wastes locally significantly and positively influenced rural waste classification and environmental protection awareness. The usual waste collection and classification improves the residents’ willingness to preserve the environment. In other words, it influences their personalities. An individual’s personality plays a significant role in enhancing their desire to collect, sort, and dispose of the wastes. This finding is supported by the authors of [28], who documented that a strong personality incorporates moral obligations, hence influencing the willingness to act. It, therefore, influences rural waste classification and environmental protection awareness among rural residents in Jiangsu.

Charging for waste disposal in the rural areas of Jiangsu significantly influenced waste classification and environmental protection awareness negatively. Imposing charges on rural waste collection induces negative feelings from the residents. This affects waste classification and environmental protection awareness adversely. Further, charging households for waste disposal services varies within and between municipalities. The marginal cost of disposing rural wastes implies that there are zero or fewer incentives for rural households to classify and dispose of the solid wastes [29,30]. Therefore, rural residents do not alter their waste classification behaviors and environmental protection awareness.

Nonetheless, age, household size, gender, monthly household income, and environmental protection rewards did not significantly influence rural waste classification and environmental protection awareness. Young rural residents should show proactiveness in acquiring scientific information compared to traditional information regarding environmental protection and its benefits [8,15,19]. Our empirical outcome depicts no significant relation between age and waste classification. Further, monthly household income was hypothesized to influence the rural waste classification and environmental protection awareness positively and significantly. In our study, there was no obvious relation between household income and rural environmental protection awareness. However, the authors of [31] concluded that households with higher income are likely to collect, classify, and dispose of wastes in the designated bins, hence protecting the environment from pollution. Further, the authors stated that the likelihood of disposing waste in the right bin increases by 6.8% per 1000 RMB increase in the local per capita income. Besides, the absence of environmental protection rewards increases the low level of environmental protection awareness and waste classification [11,12,28,29]. Also, from our findings, it was evident that there was not enough environmental protection training within the rural areas of Jiangsu. Therefore, there were no relations between environmental protection benefits with regard to rural resident training. This implies that environmental protection is not a priority compared to significant state investments.

## 5. Conclusions

Incorrect solid waste disposal has affected the rural residents’ quality of life negatively. However, currently there is no complete rural waste disposal system in the rural areas of Jiangsu province, China. Therefore, advocating for rural waste classification and environmental protection awareness is conducive to enhance the rural residents’ quality of life. In this paper, we sought to understand the different factors influencing the rural waste classification and environmental protection awareness among the rural residents of Jiangsu. We used two empirical models, the logistics regression model and ordinary least squares model, to prove our hypotheses.

In our findings, we observed that household’s level of education significantly influenced rural waste classification and environmental protection awareness regarding whether a household is composed of a village cadre; whether the village has public waste collection facilities; the distance between households and the collection facilities; whether the waste is picked, assorted, and disposed locally; and waste disposal charges. Further, regarding the significance of creating awareness about environmental protection and waste classification in rural areas, this paper suggests the following policy implications for better rural ecological protection and waste disposal. The municipality should undertake a detailed waste classification and environmental awareness training program among the residents, outlining the advantages of waste classification to the environment, residents, and country as a whole.

Even more, through the local government, the national government should increase and strengthen the sources of rural waste disposal funding. This implies that the necessary rural waste disposal facilities for classification and treatment will be constructed and sustained. Further, the authorities should understand the range within which the rural residents are willing to pay for the waste disposal. This will increase the residents’ level of participating in protecting the environment through proper waste classification and placement. Also, the authorities should outline a sound legal system to ensure that various national waste policies are implemented and followed by rural residents. The statutory regulations should be able to fill in the gaps that arise from the versed rural waste disposal problems. In the end, the rules should identify and clarify the roles of rural residents in waste classification and environmental protection.

## Figures and Tables

**Table 1:** Basic information of the four cities in Jiangsu in 2019.

City	Area (km^2^)	Total Population	GDP per Capita (yuan)
Xuzhou	11,765	8,825,600	81,138
Suqian	8524	4,925,900	62,840
Nantong	10,549	7,310,000	128,294
Changzhou	4372	4,937,900	156,390

Source: Jiangsu Statitstical Yearbook 2020.

**Table 2:** Variable description.

Variable Categories	Variable Name	Description and Assignment
Residents and family characteristics	Gender	=1 If you are Male, =0 otherwise
	Age	Actual age
	Education level	=1 if primary school and below, =2 if a middle school, =3 if technical secondary school and high school, =4 if junior college, =5 if college and above
	Household size	Total household size
	Whether there are party members or village cadres at home	=1 if yes, =0 otherwise
	Monthly household income (CNY)	=1 if less than or equal to 1000, =2 if 1001–3000, =3 if 3001–5000, =4 if 5001–7000, =5 if 7001–9000, =6 if 9000–11,000, =7 if 11,001 and above
	Are there any non-resident students at home	Residents’ awareness of environmental protection and household waste classification
Residents’ awareness of environmental protection and household waste classification	Is it necessary to carry out environmental protection work	=1 if yes, =0 otherwise
	Knowledge of garbage classification	=3 if you know it very well, =2 if you know it well, =1 if you know a little bit, =0 if you don’t know
	Is garbage classification necessary	=1 if necessary, =0 otherwise
Provision of public services	Does the village have public garbage collection facilities (garbage cans, garbage pools, etc.)	=2 if there’s a lot, =1 if there’s a lot, =0 if there’s no
	The distance between the nearest public garbage can (or trash pool) and your home	Distance (m)
	Is there someone to clean it regularly	=1 if yes, =0 otherwise
	Whether there are floating scrap vendors or people buying scrap	=1 if yes, =0 otherwise
	Whether the garbage is picked up, sorted and collected locally	=1 if yes, =0 otherwise
	Whether to charge for garbage disposal	=1 if yes, =0 otherwise
	Is there any publicity on environmental protection and waste classification	=1 if yes, =0 otherwise
Policy institutional environment	Whether there are relevant rewards for the residents who have a high degree of participation in the classification of domestic garbage	=1 if yes, =0 otherwise
	Whether to educate the residents on the behavior of destroying the village environment	=1 if yes, =0 otherwise
Waste centralized collection behavior	Clean household waste on own initiative	=1 if yes, =0 otherwise
	Just drop the waste anywhere	=1 if no, =0 otherwise
	Whether package waste and put in a designed place	=1 if yes, =0 otherwise
	Frequency of cleaning	=2 if it is twice and over, =1 if it is once a week, =0 if there’s no
Simple handling behavior	Whether do waste classification	=1 if yes, =0 otherwise
	Whether recycle and sell the parts	=1 if yes, =0 otherwise

**Table 3:** Research descriptive analysis.

Variable Name	Mean	Standard Deviation	Min	Max
Gender	0.544	0.499	0	1
Age	46.51	2.646	18	79
Education level	3.785	1.277	1	5
Household size	4.127	1.289	1	10
Whether there are party members or village cadres at home	0.498	0.454	0	1
Monthly household income(CNY)	3.874	1.819	1	7
Are there any nonresident students at home	0.359	0.48	0	1
Is it necessary to carry out environmental protection work	0.932	0.251	0	1
Knowledge of garbage classification	1.475	0.743	0	3
Is garbage classification necessary	0.794	0.405	0	1
Does the village have public garbage collection facilities (garbage cans, garbage pools, etc.)	1.511	0.546	0	2
The distance between the nearest public garbage can (or trash pool) and your home (m)	117.21	224.68	0	2000
Is there someone to clean it regularly	0.813	0.394	0	1
Whether there are floating scrap vendors or people buying scrap	0.906	0.293	0	1
Whether the garbage is picked up, sorted, and collected locally	0.362	0.481	0	1
Whether to charge for garbage disposal	0.193	0.395	0	1
Is there any publicity on environmental protection and waste classification	0.697	0.46	0	1
Whether there are relevant rewards for the residents who have a high degree of participation in the classification of domestic garbage	0.177	0.381	0	1
Whether to educate the residents on the behavior of destroying the village environment	0.691	0.462	0	1
Clean household waste on own initiative	0.93	0.311	0	1
Just drop the waste anywhere	0.52	0.50	0	1
Whether package waste and put in a designed place	1.77	0.473	0	1
Frequency of cleaning	1.96	0.201	0	1
Whether do classification	0.47	0.499	0	1
Whether recycle and sell the parts	0.82	0.382	0	1

**Table 4:** Household environmental protection awareness.

Variable	Logit Model		OLS Model	
	Regression Coefficient	The Standard Deviation	Regression Coefficient	The Standard Deviation
Gender	−0.0834	0.1626	−0.0105	0.085
Age	−0.0836	0.0885	−0.0575	0.0525
Education level	0.2427 **	0.0996	0.1143 **	0.0571
Household size	0.033	0.061	0.0222	0.03
Whether there are party members or village cadres at home	0.5239 ***	0.172	0.3241 ***	0.0847
Monthly household income	0.0123	0.0465	0.0234	0.0219
Are there any nonresident students at home	−0.021	0.1719	0.0465	0.0872
Does the village have public garbage collection facilities (garbage cans, garbage pools, etc.)	0.2952 *	0.1662	0.1678 **	0.0846
The distance between the nearest public garbage can (or trash pool) and your home	−0.0014 ***	0.0004	−0.0008 ***	0.0002
Is there someone to clean it regularly	−0.0033	0.2174	0.0192	0.1249
Whether there are floating scrap vendors or people buying scrap	0.3068	0.2615	0.2163	0.1603
Whether the garbage is picked up, sorted, and collected locally	0.8102 ***	0.1799	0.2500 ***	0.0911
Whether to charge for garbage disposal	−0.2817	0.2033	−0.1754 *	0.1037
Is there any publicity on environmental protection and waste classification	−0.0972	0.1992	−0.013	0.0985
Whether there are relevant rewards for the residents who have a high degree of participation in the classification of domestic garbage	0.2128	0.2366	0.1033	0.1204
Whether to educate the residents on the behavior of destroying the village environment	−0.1386	0.1098	−0.0028	0.1021
Constant	2.0282		2.1485	
R^2^	0.7061		0.1766	

***, **, * indicates that the significance level is 1%, 5%, 10% respectively.

**Table 5:** Research model analysis outcome of household waste classification behavior.

Regression Coefficient	Waste Collection Behavior				Simple Waste Classification Behavior			
	Logit Model		OLS Model		Logit Model		OLS Model	
	Coef	SD	Coef	SD	Coef	SD	Coef	SD
Gender	0.2858 *	0.1646	0.0799	0.0687	0.0892	0.1675	−0.0284	0.0536
Age	−0.0169	0.8742	−0.0115	0.0333	−0.0649	0.0910	−0.0254	0.0305
Education level	−0.0106	0.0962	0.0008	0.0370	0.2147 **	0.1014	0.0697 **	0.0347
Household size	−0.710	0.0629	−0.0333	0.0262	0.0242	0.0642	0.0138	0.0219
Village cadres at home	0.4647 ***	0.1745	0.1887 ***	0.0703	0.2759	0.1789	0.0939	0.0582
Monthly income	0.1073 **	0.0472	0.0368 *	0.0189	0.0140	0.0491	0.0071	0.0164
Nonresident students at home	−0.3684 *	0.1725	−0.1707 **	0.0713	−0.1040	0.1787	−0.0214	0.0600
Is environmental protection necessary	0.5522	0.3414	0.2405 *	0.1444	−0.6656 *	0.3729	−0.2020	0.1275
Knowledge of waste classification	0.2587 ***	0.1200	0.1307 **	0.0544	0.5279 ***	0.1223	0.1846 ***	0.0398
Is waste classification necessary	0.8709 ***	0.2289	0.3115 ***	0.0931	0.7039 ***	0.2430	0.2324 ***	0.0891
Available public waste collection facilities	0.6138 ***	0.1669	0.2588 ***	0.0678	−0.0964	0.1723	−0.0359	0.0552
The distance between public waste facility and your home	−0.0004	0.0004	−0.0002	0.0002	0.0010 **	0.0005	0.0003 **	0.0001
Regular waste cleaning	−0.2844	0.2276	−0.1062	0.0970	−0.5331 **	0.2445	−0.1813 **	0.0825
Whether there are floating scrap vendors	0.1534	0.2644	0.0673	0.1067	0.1572	0.2834	0.0864	0.0946
Waste collected is sorted locally	0.3680 **	0.1758	0.1003	0.0724	−0.0059	0.1792	0.0215	0.0603
Charging for waste disposal	0.0145	0.2005	0.0235	0.0807	−0.3121	0.2034	−0.1181 *	0.0683
Waste classification awareness	−0.0500	0.2013	−0.0155	0.0834	0.4020 *	0.2115	0.1053 *	0.0692
Rewards in waste classification participation	−0.2113	0.2299	−0.0356	0.0846	−0.3480	0.2407	−0.1286	0.0814
Educate residents on environmental protection behavior	0.4333 **	0.1905	0.1797 *	0.0803	0.3095	0.1947	0.1166 *	0.0616
Constant			2.8976				0.7233	
R^2^			0.1941				0.1592	

***, **, * indicates that the significance level is 1%, 5%, 10% respectively.
